# Does the frailty index applied to randomised controlled trials really measure frailty?

**DOI:** 10.1093/ageing/afaf314

**Published:** 2025-10-24

**Authors:** Rîme Bousetta, David A McAllister, Heather Wightman, Jim Lewsey, Peter Hanlon

**Affiliations:** School of Public Health, L'Université libre de Bruxelles, Brussels, Belgium; School of Health and Wellbeing, University of Glasgow, Glasgow G12 8QQ, UK; School of Health and Wellbeing, University of Glasgow, Glasgow G12 8QQ, UK; School of Health and Wellbeing, University of Glasgow, Glasgow G12 8QQ, UK; School of Health and Wellbeing, University of Glasgow, Glasgow G12 8QQ, UK; School of Health and Wellbeing, University of Glasgow, Glasgow G12 8QQ, UK

**Keywords:** frailty, frailty index, randomised controlled trials, treatment effect modification, older people

## Abstract

**Background:**

Cumulative deficit frailty indices from randomised controlled trials (RCT) are increasingly used to assess whether trial findings are applicable to people living with frailty. The aim of this paper was to examine the range and type of deficits included in these frailty indices and compare these to those from cohort studies.

**Methods:**

We identified RCTs assessing treatment effect modification using the cumulative deficit frailty index, as well as cohort studies assessing mortality risk associated with frailty, from recent systematic reviews. We extracted the deficits included in the frailty index from each RCT and cohort study. We compared the number of deficits, data sources (e.g. medical history, physical measurements, questionnaires, etc.) and physiological domain (e.g. cardiometabolic, neuro-cognitive, physical function, etc.) of the deficits from each source.

**Results:**

The number of deficits was similar between RCT frailty indices (median 41 deficits, interquartile range [IQR] 35–50) and cohort studies (median 35, IQR 31–45). Broadly similar data sources were used to identify deficits. However, in RCTs of cardiovascular conditions, cardiometabolic deficits made up a greater proportion of deficits (median 47% of included deficits, IQR 38%–51%, compared to 19%, 14%–24%, in cohort studies). Cardiovascular RCTs included fewer physical function measures (median 4% [3%–9%], compared to 16% in other RCTs of other conditions [13%–17%], 17% in cohort [13%–23%]).

**Conclusion:**

In many cardiovascular RCTs, frailty indices focus on cardiometabolic deficits rather than measures of function. These frailty indices need to be validated against outcomes important to people living with frailty before being used to inform treatment. Until then, we would emphasise caution.

## Key points

- Some randomised controlled trials use a frailty index to infer the applicability of their findings to people living with frailty.
- RCTs and observational studies use similar number of deficits and data sources to construct frailty indices.
- Cardiovascular RCT frailty indices are often dominated by cardiovascular deficits, with little assessment of function.
- Assessment of cognition or sensory function was rare in RCT frailty indices and highly variable in observational studies.
- We need to test whether narrow frailty indices such as these accurately identify frailty as seen in clinical practice.

## Introduction

How evidence about treatments should be applied to people living with frailty is a fundamental question in the care of older people. People living with frailty are, by definition, at increased of decompensation in response to physiological stressors [1, 2]. This may alter the balance of risks and benefits of an intervention. One way of answering this question is to assess whether frailty modifies the effect of treatment within randomised controlled trials (RCTs): [3, 4] i.e. whether the safety and efficacy of treatment varies depending on the frailty status of trial participants. RCTs, in which participants are randomly assigned to the intervention of interest or a comparator treatment, have clear advantages in reducing potential bias such as confounding. However, there is ongoing debate as to whether RCTs assess ‘frailty’ adequately [5–7].

The most common approach to assessing treatment effect modification by frailty is to apply a cumulative deficit frailty index (FI) to individual participant data from RCTs [8, 9]. This approach has been applied to RCTs of vaccinations, pharmacological interventions and treatment strategies for hypertension or diabetes [10–14]. A frailty index quantifies frailty as the number of age-related health deficits present within an individual, divided by the total number of deficits measured [9, 15]. Cumulative deficit frailty indices are not based on a pre-specified list of deficits. Rather, the deficits that are included are selected from the available data from a given source (e.g. a survey, cohort study, or a trial baseline assessment) provided they increase with age, are not ubiquitous, and cover a range of organ systems [9]. Some iterations of the frailty index include deficits across a wide range of physical, cognitive and functional domains and have been validated against a range of outcomes identified as important to older people (including falls, care home admission, functional independence and mortality) [16]. However, because most frailty indices are constructed from the deficits available in a given dataset the breadth and number of the available deficits can vary and the resulting index is not necessarily validated against a range of outcomes. There is variation in the specific deficits used to define frailty in different applications of the frailty index approach. This variation is the source of the current debate around the use of frailty indices in trials, which some argue rely too heavily on deficits which measure severity of the index conditions in the trial (e.g. cardiovascular deficits within a trial of hypertension or heart failure) and fail to capture broader markers of vulnerability such as physical or cognitive function [5, 6].

Whilst the frailty index approach appears robust to some variation in the deficits included, this flexibility has limits. For example, excluding entire ‘domains’ (such as cognition or physical function) reduces the performance of a frailty index as a predictor of adverse outcomes [17]. Changes in the number and selection of deficits also impact associations with mortality [18]. Also, the original developers of the frailty index make clear that where deficits are drawn from a narrow domain, it can no longer be considered a ‘frailty’ index [9]. Therefore, if the purpose is to inform the management of people living with frailty in clinical practice it is important to examine how frailty indices are constructed from RCT data. This study aims to assess what variables have been used to construct frailty indices in RCTs, and compare this to applications of frailty indices in cohort studies.

## Methods

We identified RCTs from two recent systematic/scoping reviews [19, 20]. Both of these reviews included RCTs for any condition in which frailty was assessed using any measure. Yao *et al*. included all trials assessing treatment effect modification by frailty, whilst Sun *et al.* included all trials that measured frailty before identifying those assessing treatment effect modification. Search dates were December 2023 and March 2023, respectively. From these two reviews, we identified all trials using a frailty index and included in our analysis any trial in which a frailty index was constructed based on variables collected as part of the trial. We excluded trials that used linked data to frailty indices based on electronic medical records.

We identified observational cohort studies from a systematic review assessing the association between frailty indices and all-cause mortality [21]. This review included studies in which a cumulative deficit frailty index was assessed at baseline and in which analyses assessed associations between frailty and all-cause mortality (search date July 2017). We included each of the cohort studies identified in this review. This was not intended to be a comprehensive set of cohorts assessing frailty, but rather a systematically identified sample with which to compare the RCT derived frailty indices.

For each of the RCTs and cohort studies identified, we extracted and harmonised each of the 1543 deficits used to calculate the frailty indices. Deficits were extracted verbatim from the original manuscript/Supplementary Material. We then used ChatGPT to suggest synonyms and related concepts from each of the verbatim terms (for example, ‘difficulty dressing’, ‘dressing difficulties’ and ‘requires assistance with dressing’ were harmonised to a single term: ‘difficulty dressing’). We manually reviewed each of the suggestions, correcting where necessary, to obtain a harmonised list of deficits which mapped to the original verbatim term. All extracted data, ChatGPT prompts and harmonised deficit lists are shown in the Supplementary Material and accompanying online repository.

After harmonising the deficits, we categorised the deficits by data source (medical history, laboratory/physical measurements, symptom questionnaires etc.) and also by domain/organ system. Data source was assigned based on the description in the included study. Classification by organ system was an iterative process based on inspection of the full range of deficits across the included studies which were manually allocated to organ systems (based on the Medical Subject Heading hierarchy) where appropriate as well as additional categories capturing physical function, sensory function, cognition etc. We then compared the number of deficits between the RCT frailty indices and the cohort frailty indices, visualised the specific deficits that were used across each data source, and compared the proportion of deficits within each domain between the RCTs and the cohort data.

All original and harmonised deficits along with ChatGPT prompts can be found at https://doi.org/10.5281/zenodo.16812937.

## Results

The previous reviews identified 19 different frailty indices derived from RCT baseline data (after excluding two trials that used frailty indices from linked healthcare data, excluding one study that did not provide detail of the individual deficits, and collapsing studies in two instances in which the same frailty index was used across two related trials). Twelve were assessing treatments for cardiometabolic conditions (heart failure, type 2 diabetes, hypertension, chronic kidney disease, atrial fibrillation), two were of vaccinations, one in osteoarthritis, and three focusing on older people with no specific index conditions. The frailty index was pre-specified in one of the included RCTs, whilst in the other cases each frailty index was constructed post-hoc. We identified 18 cohort studies assessing the association between a frailty index and mortality.

The median number of deficits included in the frailty index was 41 (interquartile range [IQR] 35–50) in the RCTs and 35 (IQR 35–50) in the cohort studies.

**Figure 1:**
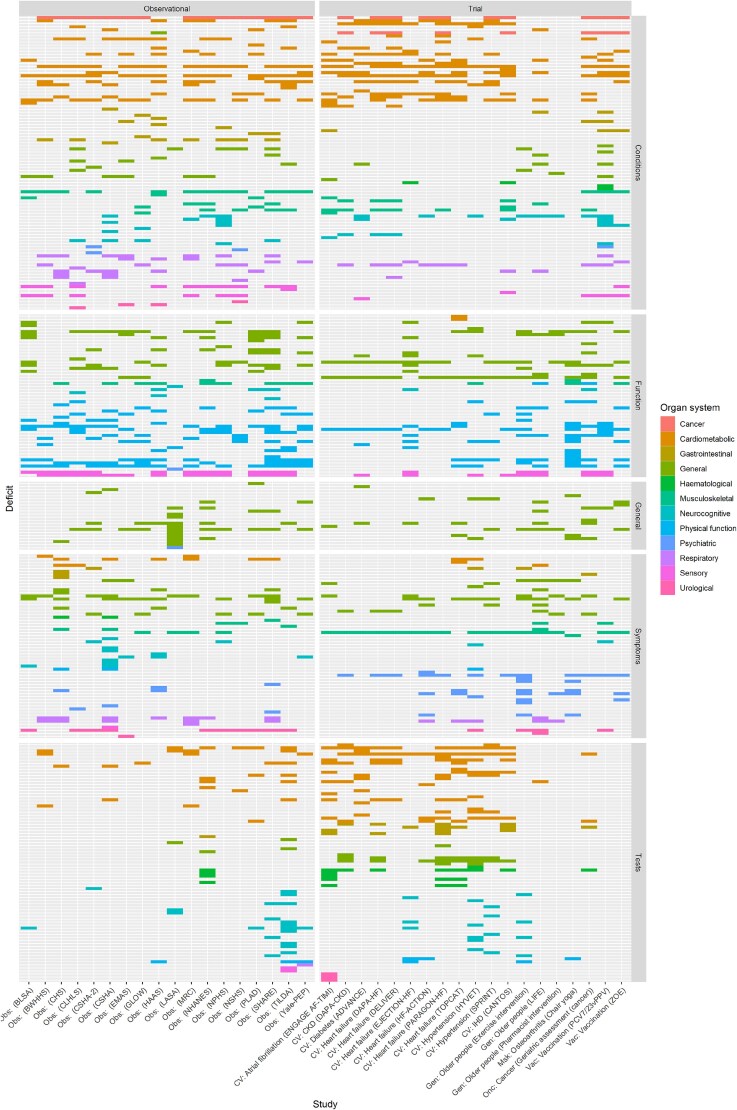
Heatmap of deficits included in each frailty index. Each study (trial or cohort) is represented by a column. Each of the harmonised concepts included as deficits are represented by a row. A version of this figure in which all deficits are labelled on the y-axis is shown in the Supplementary Material. Colour indicates the organ system of each deficit and the figure is subdivided by the type of deficit/data source (health conditions, functional measures, general, symptoms, tests).

There were 1543 different deficits which mapped to 309 unique concepts (Supplementary Material). The data sources (e.g. medical history, laboratory tests, functional questionnaires) used to identify each of the deficits are summarised in Figure 1. Both RCTs and cohort studies used deficits from a range of these data sources, although laboratory measurements were used more frequently in RCTs compared to cohort studies.

**Figure 2:**
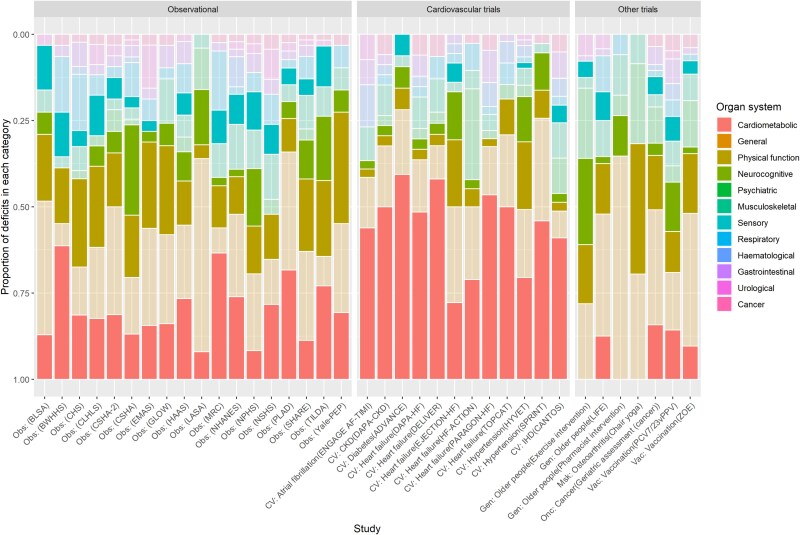
Physiological systems covered by each frailty index. This barplot shows, for each study, the proportion of deficits within the frailty index that belong to each physiological system. Systems are indicated by colour. We have emphasised cardiometabolic, physical function, neurocognitive and sensory as the domains which showed clear differences between trials and cohort studies. Full lists of all deficits within each domain are in the Appendix 1.

Figure 2 shows the proportion of deficits from each physiological domain from each study. Of note, the proportion of cardiometabolic deficits in frailty indices of the cardiovascular RCTs was considerably higher (median 47% of deficits, IQR 38%–51%) than in other RCTs (median 10%, IQR 0%–14%) or cohort studies (median 18%, IQR 14%–24%). Physical function contributed less to frailty indices in cardiovascular RCTs (median 4% of deficits, IQR 3%–9%) than in other RCTs (median 16%, IQR 13%–17%) or cohort (median 17%, IQR 13%–23%). Cognitive and sensory deficits were also notably lower in trials compared with cohort studies (Figure 2).

## Discussion

RCTs and cohort studies were broadly similar in the number of deficits they included in their frailty index and in the data sources used to identify these deficits. The most striking difference was for cardiovascular RCTs which, in many cases, included a high proportion of cardiovascular deficits, few deficits assessing physical function and none assessing cognitive or sensory function. The omission of cognitive and sensory domains was not unique to RCTs, indeed they were not included in frailty indices for many cohort studies as well. These observations have important implications for assessing the prevalence of frailty in RCTs and the efficacy and safety of treatments in people living with frailty.

The selection of deficits within RCTs may impact the assessment of how ‘frail’ or otherwise the trial population is. RCT eligibility criteria may exclude those with the most severe frailty, either explicitly or through exclusion criteria based on age, comorbidities or functional status [22]. In addition, the demands of participating in an RCT (including assessment procedures and study visits) may act as a further barrier to including people living with frailty [23]. If a frailty index in an RCT is then predominantly based on deficits that are common to trial participants (such as those related to the index condition) and does not measure deficits that may be related to exclusion from the trial (such as mobility difficulties or cognitive impairment), such a frailty index is likely to overestimate the degree of frailty in the trial population.

In general, the included cardiovascular RCTs reported similar effect estimates across levels of the frailty index with no statistically significant differences in efficacy at different levels of frailty index [20]. From this, some authors make explicit claims about the treatment of people living with frailty based on these findings, such as ‘frailty should not be a barrier to intensive blood-pressure control’ or that findings ‘should challenge any clinical reluctance to [start treatment in] patients perceived to be frail’ [24, 25]. The validity of these claims for informing clinical practice depends on the extent to which the frailty index used in these studies reflects frailty as it manifests within routine care. Ultimately, this is a question that needs to be tested (e.g. by examining whether these measures predict adverse events in a trial setting and outcomes relevant to frailty within routine care) [26]. Our findings, however, highlight important properties of RCT frailty indices that call for careful examination of how a frailty index based largely on cardiovascular deficits predicts clinical outcomes that are relevant to the care of people living with frailty.

People living with frailty are at risk of a range of adverse outcomes including falls, delirium, cognitive decline, loss of function or independence, admission to hospital or long-term care facilities and death [2, 16, 27, 28]. The defining feature of frailty is that individuals are more vulnerable to decompensation in response to (often minor) stressors that may lead to these outcomes [1]. To claim that a treatment strategy is safe and effective for people living with frailty, and likely to lead to overall benefit, relies on the assumption that people identified as ‘frail’ are genuinely at risk of the decompensation that leads to these outcomes. We would argue that the frailty index as implemented in RCTs needs to be (or have been) validated against these outcomes.

Our study strengths include identification of RCTs from recent systematic reviews and a detailed extraction and categorisation of the deficits used in each frailty index. A limitation to our study is that we relied on previously published (albeit recent) systematic reviews to identify eligible trials and cohort studies. Whilst this gave a broad range of RCTs (given the broad review inclusion criteria) this may not be comprehensive and may miss more recent studies. Another limitation is that whilst we were able to describe differences in the proportion of included deficits, we were not able to assess the impact of these differences on the performance of each frailty index. We would argue that this is an important avenue for future research. If it were that these frailty indices perform similarly with respect to a wide range of outcomes important to older adults, then their use in RCTs may provide reassurance of the efficacy and safety of certain treatments in these populations. However, if the exclusion of certain domains leads to the under-estimation of risks of outcomes such as falls or delirium, this would call into question the ability of these analyses to assess the applicability of these RCTs to people living with frailty.

Until the impact of deficit selection in RCTs is carefully examined, we would emphasise caution in judging the applicability of trials to people living with severe frailty based on frailty indices with limited assessment of function. We cannot assume that these narrower assessments of frailty adequately capture the broad risks experienced by people living with frailty such as falls, delirium and loss of independence. Our findings indicate that work is urgently needed to establish if narrower frailty indices demonstrate adequate prediction of these outcomes. If they do, then their use in assessing the safety and efficacy of treatment for people living with frailty could be valuable. However, until then, we would argue that claims based on these indices suggesting safety and overall benefits of treatment across the frailty spectrum may be premature and are unlikely to be valid in people with more advanced or severe frailty.

## Supplementary Material

aa-25-1892-File002

## Research Data Transparency and Availability:

All original and harmonised deficits along with ChatGPT prompts can be found at https://doi.org/10.5281/zenodo.16812937.
