# Supplementary material for: Does the frailty index applied to randomised controlled trials really measure frailty?
Source: Age Ageing. 2025 Oct 24;54(10):afaf314. doi: 10.1093/ageing/afaf314 (PMC12551376; doi:10.1093/ageing/afaf314)
Supplement: aa-25-1892-File002 [file aa-25-1892-file002.docx]

**Implementation of Blood Flow Restriction training in the Acute Geriatric Unit**

Contents list:

**Appendix 1, page 2**

**Appendix 2, page 5**

**Appendix 3, page 6**

**Appendix 4, page 7**

**Appendix 5, page 8**

**Appendix 6, page 8**

**Appendix 1**

**Full Materials and Methods**

**Study design and participants**

This study was a retrospective observational study with an active control group. Between 12 February and 6 September 2024, blood flow restriction (BFR) training was integrated into routine physiotherapy on one of two comparable acute geriatric unit (AGU) at Ghent University Hospital. The second one continued with standard physiotherapy alone and served as the control group. Patient allocation to AGUs occurred based on bed availability upon admission. For this study, we included all patients from the two AGUs who received standard physiotherapy and did not meet any of the predefined contraindications for BFR training. These contraindications included: 1) inability to walk for at least one minute (with or without walking aid), 2) hospitalization for orthopedic surgery involving the upper or lower limbs, 3) inability to perform strength training due to severe dyspnea at rest or need for oxygen therapy, 4) unstable hypertension (resting systolic blood pressure >180 mmHg) or tachycardia (resting heart rate >100 bpm), 5) unhealed skin grafts on the lower limbs, 6) increased intracranial pressure, 7) active infection of the lower limbs, 8) significant lower limb swelling due to recent surgery or lymphatic dysfunction, 9) presence of dialysis access in the lower limbs, 10) open fractures in the lower limbs, 11) large soft tissue hematomas in the lower limbs, 12) peripheral artery disease with ischemic rest pain, gangrene or ulcerations, 13) peripheral polyneuropathy with complete numbness and/or ulcerations, 14) accommodation in an isolation room. The study protocol was approved by the Ethics Committee. As the study involved secondary analysis of routinely collected clinical data, the requirement for written informed consent was waived. Patients were informed via the hospital’s patient portal about the use of their data for research purposes.

**Exposure of Interest**

All eligible patients participated in a standardized physiotherapy program on weekdays, excluding weekends and public holidays (**Figure 1**). Each session commenced with baseline assessments, including blood pressure and heart rate measurements. In the BFR group, limb occlusion pressure (LOP) was additionally measured before exercise. The session structure included a supervised walking bout of up to 10 minutes or until voluntary fatigue—performed with BFR in the BFR group and without in the control group. This was followed by a 15-minute unsupervised aerobic exercise segment (without BFR), and a 5-minute supervised strength and balance training component tailored to individual ability. A second supervised walking bout (again up to 10 minutes), conducted with or without BFR according to group assignment, concluded the session. For the BFR group, pneumatic cuffs (SmartCuffs PRO, SmartTools) were positioned on the proximal thighs and inflated to 60% of the individually determined LOP whilst standing. Cuffs were deflated between walking segments and for all other exercises. Cuff size (small, medium, large) was selected visually based on thigh circumference. For the 15-minute unsupervised aerobic segment, patients used one of three machines: MOTOmed loop.la (RECK MOTOmed), Mag Bike 108-B (DKN Technology), or NuStep® T4r recumbent cross-trainer (NuStep). The physiotherapist adjusted the workload based on patient tolerance. During the supervised strength and balance training, equipment included: 1) the Gerobank V system combining leg press and low row exercises or used separately, 2) ankle and wrist weights (0.5–2 kg) and resistance bands (0.5–2.5 kg) for upper or lower limb exercises, 3) dumbbells (0.5–3 kg) for upper limb exercises, 4) the Airex balance pad for balance exercises. Exercises were chosen based on individual capacity and clinical judgment.

**Outcomes of Interest**

Outcomes were derived from routine physiotherapy assessments. They were grouped into three primary domains: feasibility, safety and effectiveness.

**Feasibility**

Feasibility of BFR training was evaluated using three components: adherence, satisfaction and practicality, following the framework proposed by Bowen *et al.* and Dhokia *et al.* [32,33].

Adherence was calculated as the number of completed physiotherapy sessions divided by the number of scheduled sessions during the first week of hospitalization. Sessions missed due to physiotherapist unavailability, overlapping medical procedures, or temporary medical contraindications (as determined by the attending geriatrician or physiotherapist) were excluded from the denominator. A session was considered completed if at least one of the two scheduled walking bouts (with BFR for the intervention group, without BFR for the control group) was performed.

Satisfaction was evaluated both qualitatively and quantitatively. The number of patients in the BFR group who reported pain or discomfort due to cuff application during the measurement procedure or physiotherapy sessions was recorded. Additionally, pain collected following the first supervised walking session was routinely assessed using the Numeric Pain Rating Scale (NPRS), ranging from 0 ("no pain") to 10 ("worst imaginable pain").

Practicality was assessed by documenting operational challenges, including the number of failed limb occlusion pressure (LOP) assessments during the measurement procedure and occurrences of cuff loosening while walking due to misplacement or incorrect sizing. To determine whether daily LOP reassessment is necessary in clinical practice, intra-individual variability in daily LOP assessments was also calculated.

**Safety**

Adverse events were identified through the daily physiotherapy logs and categorized manually using the Common Terminology Criteria for Adverse Events (CTCAE), version 5.0. The CTCAE was selected for its applicability beyond pharmacological interventions, allowing classification of adverse events related to physical therapy and exercise [34,35].

**Effectiveness**

Effectiveness was evaluated across two domains: physical function and endurance.

Standardized evaluations of physical function (grip strength and Short Physical Performance Battery or SPPB) were performed within 72 hours of hospital admission and again approximately one week after initiating physiotherapy (±2 days), but only in patients who completed at least two physiotherapy sessions and remained hospitalized (N=54). Upper body strength was quantified by grip strength using a dynamometer. Lower extremity physical function was assessed using the Short Physical Performance Battery (SPPB; score range 0–12) [36]. The SPPB consists of three subtests: (1) static balance evaluation (score range 0–4) assessed by the ability to hold three progressively more difficult standing positions (feet together, semi-tandem, tandem) for up to 10 seconds each; (2) usual gait speed via the 3-meter walk test (3MWT); and (3) lower limb strength evaluated by the Timed Chair Stand test (TCS), which requires rising from a chair five times as quickly as possible without using the armrests.

Endurance-related outcomes were available for all patients who initiated physiotherapy (N=123), as these data were routinely documented after the first supervised walking session and during subsequent sessions. Endurance was assessed by walking duration and perceived exertion, rated using the Borg Rating of Perceived Exertion scale (Borg RPE; range 0–10).

**Statistical Analysis**

Descriptive statistics were reported as means with standard deviations (SD) for continuous variables and as frequencies with percentages for categorical variables. Differences between the BFR and control group in the descriptives, feasibility (excluding NPRS) and safety outcomes were analyzed using the Wilcoxon rank-sum test for continuous variables due to non-normal distributions, and Pearson’s Chi-squared test (expected cell counts ≥5) or Fisher’s exact test (expected count <5) for categorial variables. To evaluate effectiveness outcomes and NPRS, regression models were built: linear regression models for grip strength and SPPB scores, mixed-effects linear models accounting for within-subject correlations for walking duration, Borg RPE and NPRS. Tobit regression modifications were applied for linear regression outcomes with clear floor or ceiling effects (SPPB, SPPB balance subscore and Timed Chair Stand). Given the 10-minute maximum for walking duration, in addition to the mixed-effects linear model, sensitivity analyses were conducted using three models that explicitly account for right-censoring: a Tobit mixed-effects model, a mixed-effects Cox proportional hazards model, and an Accelerated Failure Time (AFT) model [37]. In all regression models, the interaction term between group allocation (with the BFR group as reference) and the number of physiotherapy sessions was the primary variable of interest, representing the differential effect of session number on outcomes between the BFR and control groups.

For continuous outcomes, standardized effect sizes (Cohen’s *d*) were also calculated. In the regression analyses, predicted standardized effect sizes were calculated based on a fixed number of sessions (i.e., 8), assuming an average hospital stay of 10 days, which typically includes 2 weekend days. These estimates were derived using the model parameters [38]. All models were adjusted for age, sex, time, number of physiotherapy sessions and baseline values of the respective outcome. A two-sided *P*-value <0.05 was considered statistically significant. Analyses were performed using R version 4.4.0 (R Foundation for Statistical Computing, Vienna, Austria).

**Appendix 2**

**
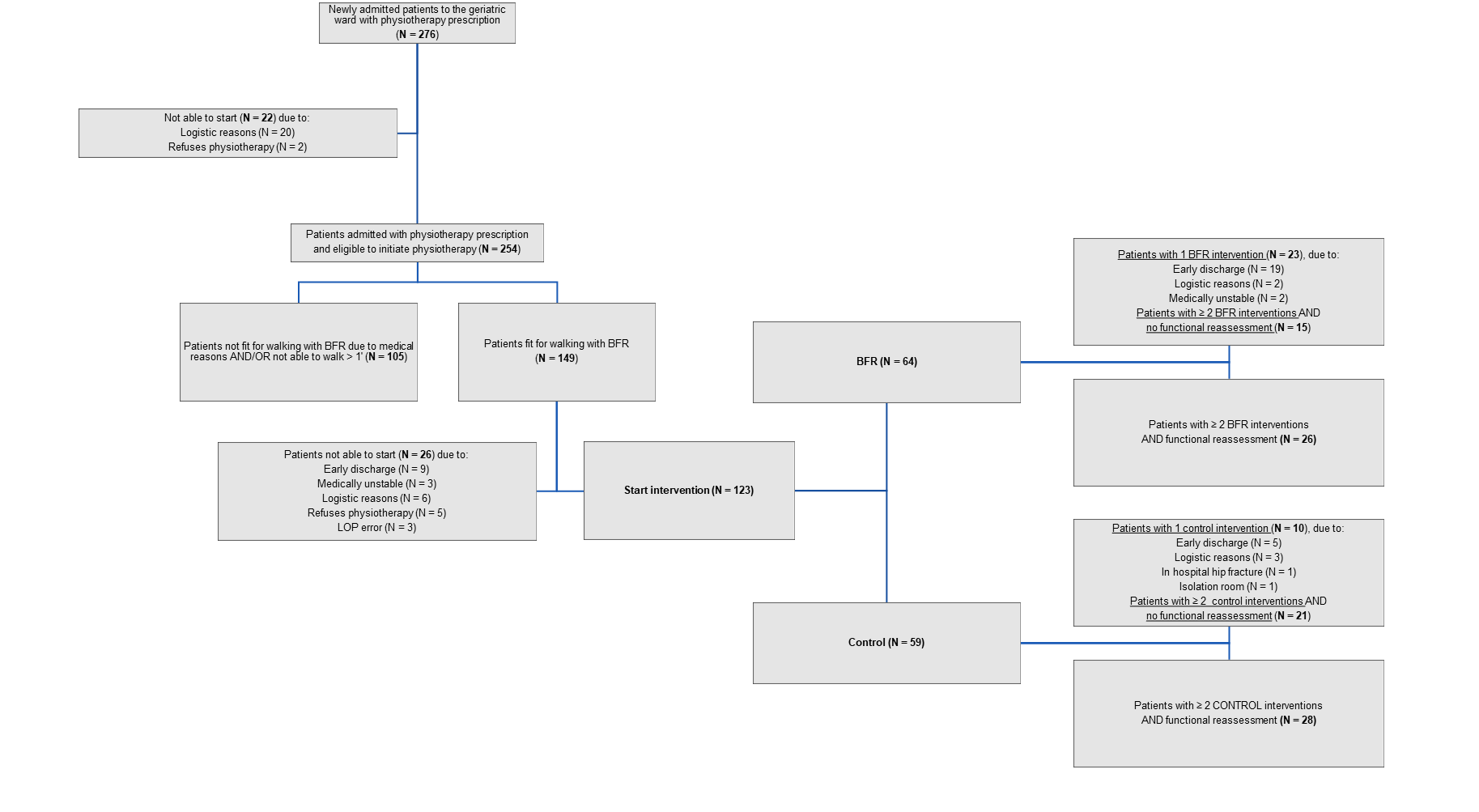
**

**Appendix 3**

Appendix 3. Baseline characteristics of the physical function subcohort. BFR (Blood Flow Restriction group); CON (control group); SARC-F (Strength, Assistance with walking, Rise from a chair, Climb stairs, and Falls questionnaire); SPPB (Short Physical Performance Battery); 3MWT (Three Meter Walking Test); SD (Standard Deviation); N (Number of patients).

| **Baseline characteristics** | | | | |
| --- | --- | --- | --- | --- |
| **Characteristic** | **Overall** | **BFR** | **CON** | **p-value^2^** |
|  | N=54^1^ | N=26^1^ | N=28^1^ |  |
| Age (yr) | 83.85 (5.99) | 84.19 (6.58) | 83.54 (5.48) | 0.6 |
| Sex |  |  |  | 0.3 |
| Female | 36 (67%) | 19 (73%) | 17 (61%) |  |
| Male | 18 (33%) | 7 (27%) | 11 (39%) |  |
| Place of living | |  |  | 0.1 |
| Community | 41 (76%) | 23 (88%) | 18 (64%) |  |
| Assisted Living | 7 (13%) | 2 (7.7%) | 5 (18%) |  |
| Nursing home | 6 (11%) | 1 (3.8%) | 5 (18%) |  |
| SARC-F (0-10) | 4.87 (3.04) | 4.41 (3.19) | 5.30 (2.90) | 0.3 |
| (Not able due to cognitive impairment) | 9 | 4 | 5 |  |
| Hand grip strength (kg) | 19.05 (7.93) | 17.87 (5.75) | 20.15 (9.49) | 0.4 |
| SPPB Balance test (0-4) | | |  | 0.8 |
| 0 | 12 (22%) | 4 (15%) | 8 (29%) |  |
| 1 | 8 (15%) | 4 (15%) | 4 (14%) |  |
| 2 | 17 (31%) | 8 (31%) | 9 (32%) |  |
| 3 | 9 (17%) | 5 (19%) | 4 (14%) |  |
| 4 | 8 (15%) | 5 (19%) | 3 (11%) |  |
| SPPB 3MWT (s) | 9.35 (7.92) | 8.91 (5.82) | 9.76 (9.57) | 0.9 |
| Usual gait speed (m/s) | 0.46 (0.23) | 0.45 (0.21) | 0.47 (0.25) | 0.9 |
| Used a walking aid during 3MWT | 32 (59%) | 16 (62%) | 16 (57%) | 0.7 |
| SPPB 5 chair stands | |  |  | 0.1 |
| > 60 sec or unable | 32 (59%) | 15 (58%) | 17 (61%) |  |
| 16.70-60 sec | 17 (31%) | 11 (42%) | 6 (21%) |  |
| 13.70-16.69 sec | 3 (5.6%) | 0 (0%) | 3 (11%) |  |
| 11.20-13.69 sec | 2 (3.7%) | 0 (0%) | 2 (7.1%) |  |
| < 11.20 sec | 0 (0%) | 0 (0%) | 0 (0%) |  |
| SPPB (0-12) | 4.11 (2.52) | 4.31 (2.33) | 3.93 (2.72) | 0.5 |
| ^1^ Mean (SD); n (%) | | | | |
| ^2^ Wilcoxon rank sum test; Pearson’s Chi-squared test; Fisher’s exact test | | | | |

**Appendix 4**

Appendix 4. Baseline characteristics of the patients that did not receive a physical function reassessment after 1 week (N=69). BFR (Blood Flow Restriction group); CON (control group); SARC-F (Strength, Assistance with walking, Rise from a chair, Climb stairs, and Falls questionnaire); SPPB (Short Physical Performance Battery); 3MWT (Three Meter Walking Test); SD (Standard Deviation); N (Number of patients).

| **Baseline characteristics** | | | | |
| --- | --- | --- | --- | --- |
| **Characteristic** | **Overall** | **BFR** | **CON** | **p-value^2^** |
|  | N=69^1^ | N=38^1^ | N=31^1^ |  |
| Age (yr) | 84.88 (5.28) | 85.39 (4.73) | 84.26 (5.91) | 0.4 |
| Gender |  |  |  | 0.7 |
| Female | 31 (45%) | 18 (47%) | 13 (42%) |  |
| Male | 38 (55%) | 20 (53%) | 18 (58%) |  |
| Place of living |  |  |  | 0.2 |
| Community | 55 (80%) | 30 (79%) | 25 (81%) |  |
| Assisted Living | 8 (12%) | 3 (7.9%) | 5 (16%) |  |
| Nursing home | 6 (8.7%) | 5 (13%) | 1 (3.2%) |  |
| SARC-F (0-10) | 4.23 (2.68) | 4.87 (2.67) | 3.50 (2.53) | 0.1 |
| (Not able due to cognitive impairement) | 13 | 8 | 5 |  |
| Hand grip strength (kg) | 21.77 (7.72) | 21.25 (6.74) | 22.41 (8.85) | 0.7 |
| SPPB Balance test (0-4) |  |  |  | 0.9 |
| 0 | 12 (17%) | 7 (18%) | 5 (16%) |  |
| 1 | 12 (17%) | 7 (18%) | 5 (16%) |  |
| 2 | 24 (35%) | 13 (34%) | 11 (35%) |  |
| 3 | 8 (12%) | 3 (7.9%) | 5 (16%) |  |
| 4 | 13 (19%) | 8 (21%) | 5 (16%) |  |
| SPPB 3MWT (s) | 7.51 (6.90) | 7.87 (7.36) | 7.08 (6.38) | 0.2 |
| Usual gait speed (m/s) | 0.53 (0.22) | 0.50 (0.22) | 0.56 (0.23) | 0.2 |
| Used a walking aid during 3MWT | 32 (46%) | 21 (55%) | 11 (35%) | 0.1 |
| SPPB 5 chair stands |  |  |  | 0.8 |
| > 60 sec or unable | 35 (51%) | 20 (53%) | 15 (48%) |  |
| 16.70-60 sec | 28 (41%) | 16 (42%) | 12 (39%) |  |
| 13.70-16.69 sec | 5 (7.2%) | 2 (5.3%) | 3 (9.7%) |  |
| 11.20-13.69 sec | 1 (1.4%) | 0 (0%) | 1 (3.2%) |  |
| < 11.20 sec | 0 (0%) | 0 (0%) | 0 (0%) |  |
| SPPB (0-12) | 4.55 (2.47) | 4.32 (2.41) | 4.84 (2.56) | 0.4 |
| ^1^ Mean (SD); n (%)  ^2^ Wilcoxon rank sum test; Pearson’s Chi-squared test; Fisher’s exact test | | | | |

**Appendix 5**

**Appendix 5**. Sensitivity analyses for walking time outcome, after adjustment for age, sex, time, number of physiotherapy sessions and baseline walking time (N=123). For the Cox model, values <1 indicate a lower hazard to stop walking before 10 minutes (longer walking duration). The Session × Group interaction term (β) reflects the average change in walking time (seconds) per session for each group (Tobit) or multiplicative change in expected walking time (AFT). Estimate (mean predicted values after 8 sessions); CI (confidence interval); ꞵ (interaction term).

| Model | BFR group | | Control group | | Session * Group Interaction effect | | | | Model Fit |
| --- | --- | --- | --- | --- | --- | --- | --- | --- | --- |
|  | Estimate | 95% CI | Estimate | 95% CI | Effect size after 8 sessions  (Cohen’s *d*) | ꞵ | (95% CI) | *P*-value | ^1^ Marginal R² / Conditional R²  ² Nagelkerke's R² |
| GLMM | 473.24 | 424.82–527.18 | 368.12 | 337.57–401.44 | 1.24 | 0.95 | (0.92–0.97) | <0.001 | 0.568 / 0.662^1^ |
| Tobit | 445.35 | 413.68–477.02 | 356.84 | 330.74–382.94 | 1.27 | -18.05 | (-26.08–-10.02) | <0.001 | 0.674 / 0.742^1^ |
| AFT | 530.55 | 487.53–577.35 | 454.33 | 422.97–488.02 | 0.44 | 0.95 | (0.93–0.98) | <0.001 | 0.525² |
| Cox | 0.56 | 0.36–0.86 | 1.11 | 0.82-1.52 | - | 1.19 | (1.04–1.35) | 0.010 | - |

**Appendix 6**

**Appendix 6**. Walking time outcomes between number of physiotherapy sessions and allocated groups (BFR group as reference), after adjustment for age, sex, time, number of physiotherapy sessions and baseline physical function. Estimate (mean predicted values after 8 sessions); CI (confidence interval); ꞵ (interaction term).

| Outcomes | BFR group | | Control group | | Session * Group Interaction effect | | | | Model Fit |
| --- | --- | --- | --- | --- | --- | --- | --- | --- | --- |
|  | Estimate | 95% CI | Estimate | 95% CI | Effect size after 8 sessions  (Cohen’s d) | ꞵ | (95% CI) | P-value | ^1^ Marginal R² / Conditional R² |
| Endurance (N=54) |  |  |  |  |  |  |  |  |  |
| Walking time (s) | 446.59 | 387.05–515.28 | 361.48 | 321.18–406.82 | 1.08 | 0.95 | (0.91–0.99) | **0.020** | 0.572 / 0.713^1^ |
